# Can Viewing Nature Through Windows Improve Isolated Living? A Pathway Analysis on Chinese Male Prisoners During the COVID-19 Epidemic

**DOI:** 10.3389/fpsyt.2021.720722

**Published:** 2021-11-22

**Authors:** Hansen Li, Xing Zhang, Chengming You, Xin Chen, Yang Cao, Guodong Zhang

**Affiliations:** ^1^Key Lab of Physical Fitness Evaluation and Motor Function Monitoring of General Administration of Sports of China, Institute of Sports Science, College of Physical Education, Southwest University, Chongqing, China; ^2^Department of Basketball and Volleyball, Chengdu Sport University, Chengdu, China; ^3^National Forestry and Grassland Administration Key Laboratory of Forest Resources Conservation and Ecological Safety on the Upper Reaches of the Yangtze River, Sichuan Province Key Laboratory of Ecological Forestry Engineering on the Upper Reaches of the Yangtze River, Long-Term Research Station of Alpine Forest Ecosystems, Institute of Ecology and Forestry, Sichuan Agricultural University, Chengdu, China; ^4^Department of Sport and Health Sciences, Technical University of Munich, Munich, Germany; ^5^Clinical Epidemiology and Biostatistics, School of Medical Sciences, Örebro University, Örebro, Sweden; ^6^Unit of Integrative Epidemiology, Institute of Environmental Medicine, Karolinska Institutet, Stockholm, Sweden

**Keywords:** nature exposure, isolated, prison, life satisfaction, well-being

## Abstract

Nature exposure is known to promote life satisfaction and well-being, and indirect exposure through windows is likely to benefit isolated populations. However, whether such type of exposure can benefit prisoners, the extremely isolated population, is unknown. In the current study, we investigated 326 male prisoners from three prisons in southwest China. Psychological variables including depression, anxiety, loneliness, distress tolerance, life satisfaction, and well-being were measured using the Patient Health Questionnaire-9 (PHQ-9), Generalized Anxiety Disorder Scale (GAD-7), short-form UCLA Loneliness Scale (ULS-6), Distress Tolerance Scale (DTS), Satisfaction with Life Scale (SWLS), and 5-item World Health Organization Well-Being Index (WHO-5), respectively. Structural equation modeling was employed to identify the pathways from the visibility of nature through windows to prisoners' life satisfaction and well-being. Our results demonstrated that visibility of nature promoted the frequency and duration of viewing nature through windows. The frequency directly affected well-being, but the duration did not effectively affect any measured variables. The visibility of nature enhanced life satisfaction mainly *via* direct effects but enhanced well-being mainly *via* indirect effects. Regarding the indirect pathways, the visibility of nature increased distress tolerance and thus reduced loneliness and mental health problems. The reduced mental health problem, in turn, promoted life satisfaction and well-being. Our findings suggest that nature exposure through windows is effective in enhancing prisoners' life satisfaction and well-being. The policymaker may need to consider nature-based solutions such as indirect nature exposure in prions to benefit isolated populations.

## Introduction

Historically, prisons are isolated places where retribution and punishment take priority over rehabilitation (1). However, in recent years, the rehabilitation of prisoners has gradually become an essential concern due to its connection with social stability. Specifically, the prison population has exploded worldwide, resulting in more severe health problems in prison systems (2). Given that most prisoners are eventually released into the community, these floating populations from prisons to the main society may be potential threats for the public if they have health and behavioral problems. Studies suggest that mental health conditions of prisoners can affect their chance of recidivism during post-incarceration, which underlines the importance of rehabilitation of prisoners in prisons, especially mental healthcare (1, 3, 4).

During the COVID-19 pandemic, prisoners have suffered from higher risks of infection due to overcrowding, poor ventilation, and close habitation (5, 6). Over the past year, prison visits and work were frequently suspended, making life in prison more restricted (7). Most prisoners stayed in small cells for more than 20 h a day (8). Infected prisoners were quarantined alone for dozens of days, which raised the concern of exacerbated mental health conditions, such as anger, depression, psychosis, self-harm, and suicide (9–13). Even worse, routine services were usually withdrawn or disturbed in many prisons due to the risk of infection, e.g., mental health services were reduced due to the absence of prison staff, which might aggravate mental health problems during this special period (14–16). In China, infections were relatively well-controlled in prisons during the epidemic. In the first 3 months of the epidemic, only three Chinese prisons reported cases of infection. Those infections were controlled in a timely fashion, and no patient died (17). In most Chinese prisons, complete isolation only lasted for a couple of days at the epidemic outbreak, and most routines have remained. Nevertheless, some events were restricted, such as prison visits and group recreational activities. In addition, due to access restrictions for prison staff, prison services were reduced. These changes might cause negative impacts on Chinese prisoners' physical and mental health.

To maintain prisoners' health during the epidemic, prison staff have used some methods such as puzzles and coloring tasks to reduce the risk of mental health problems (15). In recent years, nature exposure has been advocated as a promising strategy for mental health improvement in prisons (1). Solutions such as planting works and nature images were found to help improve prisoners' emotional outcomes (18–20). Nevertheless, relevant attempts are still insufficient due to the inconvenience of nature exposure in prisons. Unlike the general public, who can freely access ideal greenspaces, prisoners can barely have complete and frequent contact with nature. Besides, since most prisoners have spent much time indoors during the pandemic, a green view through a window may be their only chance to connect to nature. Based on a recent study, viewing greenspaces through windows might promote the mental health of isolated people during the epidemic (21), which implies an option for prisoners to maintain mental health, especially for those who are quarantined alone due to infection. Hence, we carried out this study based on the Chinese prison system to investigate:

(1) How does the window view of nature influence prisoners' behaviors in nature exposure through windows?
(2) How does the window view of nature influence prisoners' life satisfaction and well-being directly and indirectly?

## Conceptual Framework of Exposure-Response Pathways

### Visibility of Nature: Nature Exposure Behaviors

Windows can contribute to indirect nature exposure (22, 23). According to previous studies, the visibility of nature is a fundamental dimension for nature exposure through windows (21, 22), which may be affected by neighborhood greening rate and the geographical location of windows. Theoretically, the visibility of nature may regulate the frequency and duration of viewing nature through windows because a window with a better view of nature may attract people to make more visual contact with greenness outside. In direct nature exposure, the dose of exposure is an essential factor to predict the benefits of nature exposure, which can be determined by the frequency and duration of nature visits (21, 24, 25). Therefore, the frequency and duration may also regulate the benefits of indirect nature exposure. However, these two factors were rarely considered in previous studies concerning nature exposure through windows, and the relationships among these dose-response factors are yet to be investigated.

### Nature Exposure Through Window: Mental Health Problems

Nature exposure has shown great potential in improving mental health, particularly in coping with mental health problems such as depression and anxiety (22, 26, 27). A recent study has also confirmed the same mental health benefits in nature exposure through windows (21). Notably, this study has also underlined that the visibility of greenspace through windows may alleviate the sense of loneliness. This finding has provided evidence for an emerging research topic of reducing loneliness by greenspace (28). For prisoners, loneliness is a common emotional issue (29–32), and it may exacerbate depression and anxiety symptoms (33). Therefore, it is necessary to examine the effectiveness of window views in relieving prisoners' loneliness.

On the other hand, exposure to nature may regulate stress, thus enhancing tolerance to negative emotions (34, 35), which may further reduce the risk of depression and anxiety (36, 37). Based on the similar benefits observed in direct and indirect nature exposure, it is reasonable to hypothesize that nature exposure through windows may also facilitate prisoners' mental health by reducing their sense of loneliness as well as enhancing distress tolerance.

### Nature Exposure Through Window: Mental Health Problems—Life Satisfaction and Well-Being

Viewing nature through windows has been proved to promote life satisfaction and well-being (38, 39). According to previous studies, life satisfaction and well-being are negatively affected by mental health problems (40, 41). Although the above evidence implies that nature exposure may help prisoners cope with mental problems, the effectiveness of nature exposure through window views has not been confirmed. Prisoners are highly restricted and isolated individuals. Electronic devices such as mobile phones and personal computers are not allowed in most prison systems. Thus, prisoners may receive much less impact from networks and media during the epidemic. Moreover, the sedentary lifestyle is reported to threaten prisoners' health because prisoners usually spend much more time indoors than non-prisoners (42). These conditions may make the prisoners' responses to the window views different from those of the general public.

### Direct Acyclic Graph of the Conceptual Framework

**Figure 1:**
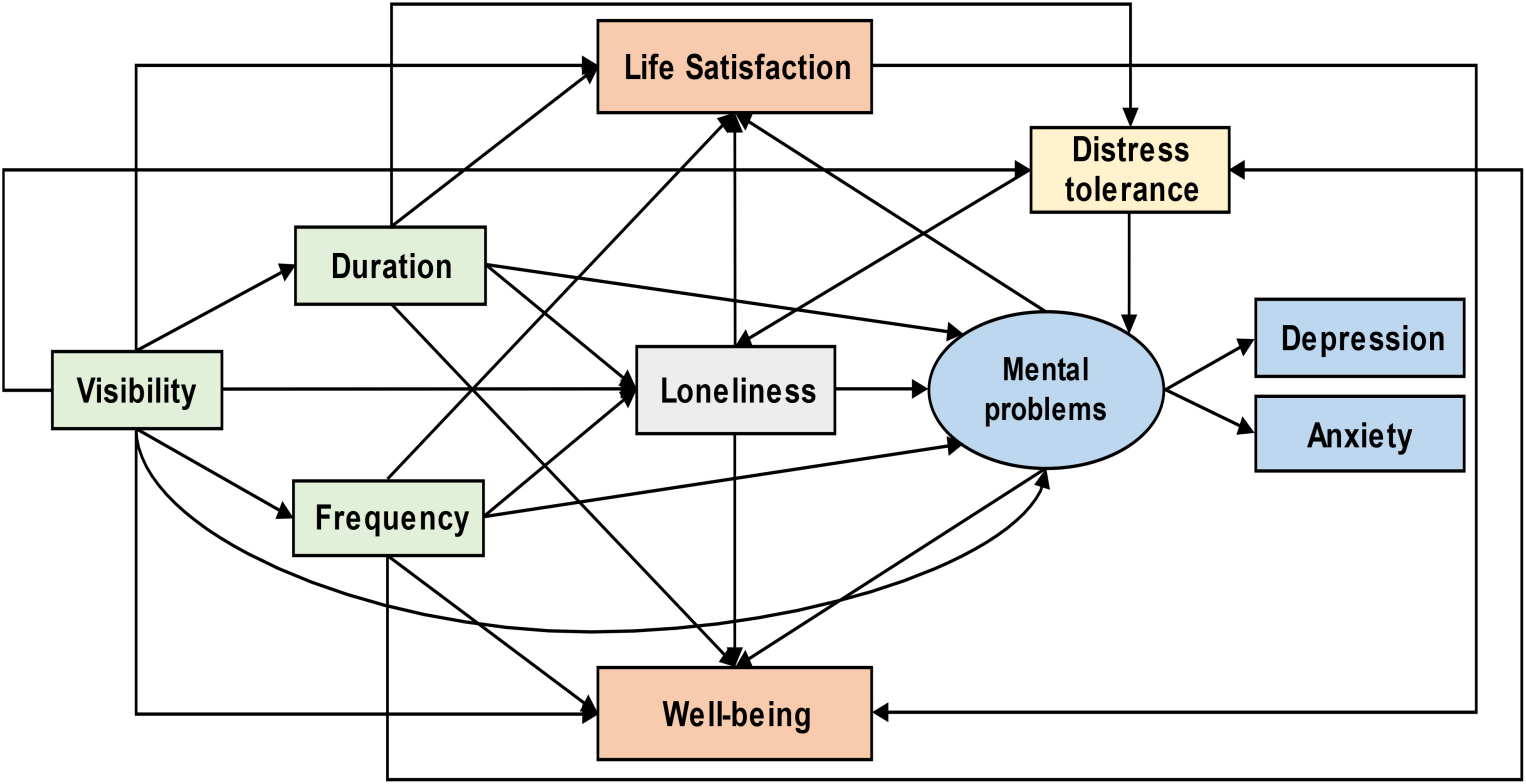
Conceptual framework of nature exposure through window: Prisoners' life satisfaction and well-being.

The conceptual framework based on the above theoretical pathways was shown in the direct acyclic graph (DAG) (Figure 1). The nature exposure through windows was measured using (1) visibility of nature, (2) duration, and (3) frequency of nature exposure through windows. We hypothesized that visibility of nature might promote the duration and frequency of nature exposure through windows; the three factors would directly promote life satisfaction and well-being and indirectly affect them *via* mediators including distress tolerance, loneliness, and mental health problems. Previous studies have suggested that life satisfaction may indicate well-being (43–45). Therefore, we also conjectured a casual directionality from life satisfaction to well-being.

## Materials and Methods

### Study Design and Population

We conducted a cross-sectional study in the southwest part of Sichuan province, China. Three prisons for males were included in the investigation. Due to the COVID-19 epidemic, prisons in China refuse any visitors. Therefore, the investigation was remotely operated with the aid of prison officers, and the collaborative study complied with the rules and policies of the prison system. According to relevant Chinese laws, forcing prisoners to participate in involuntary activities is illegal and will receive severe punishment from the Chinese National Department of Justice. Therefore, the prison staff underlined that the participation in the survey must be completely voluntary, and the final sample size and answer quality could not be predicted in advance. In addition, we required the prison staff to protect participants' privacy and not use the collected questionnaires without permission.

Before the formal investigation, the prison officers informed the prisoners about this investigation in their routine meetings. The theme of the investigation was introduced as environmental health promotion in prison. The prisoners were told that participation was voluntary, and there would be neither punishment for not participating nor consequences for their answers during the investigation.

Labor education is a Chinese policy that aims to maintain the sociality of prisoners and help them develop job skills for post-incarceration (as most prisoners don't have a substantial job before incarceration) (46). As a reward, prisoners are paid with average local remuneration for the same work, and prisoners will receive certificates of vocational skills if they can pass relevant tests. Given that the investigation would take up the prisoner's time for the labor education, we compensated them money equal to their daily salaries, and the research fund covered the expenses (SWU1909025).

A digital questionnaire was sent to prison officers and then printed in the prisons. Prison staff helped distribute and collect questionnaires. All questionnaires were self-reported by the participants and sealed in envelopes as they were collected. The investigation regulators agreed not to check the answers of the questionnaires and also not to enquire or discuss with investigated prisoners their answers and opinions concerning the questionnaires. No coercion existed in the study, and all the prisoners participated in the survey voluntarily. All the data were manually processed by the research team, and no prison staff was involved.

A consent form was attached with the questionnaire, and the participants would read and sign the form if they volunteered to participate in the investigation. The consent included a description of the items and our research purpose, but the research questions were concealed. In addition, the participants were informed that they were free to quit before or during the investigation. The consent also included a description of the purpose behind the data as well as privacy protection. In the end, the consent indicated the potential benefits and compensation of the investigation (fund compensation and the possibility of improving the prison cells).

The questionnaires were collected on March 3. The study was approved by and under supervision from the Ethics Review Board of Southwest University.

### Measurements of Nature Exposure

The visibility of nature and frequency and duration of viewing nature through windows were measured using the questions in Table 1.

**Table 1:** Questions for nature exposure through window.

**Dimensions**	**Question**	**Answer scale**
Visibility	How much greenspace can you see *via* the window of your room?	(1) Invisible (2) A bit (3) Some (4) Much
Frequency	How many times a day do you usually view the green space through window on average?	Give an estimated number
Duration	How long per time on average? (viewing greenspace through window)	Give an estimated number in minute

### Prisoners' Characteristics

Due to the prison system's policy, only limited information on the prisoners' demographic characteristics and activities was allowed for the investigation. After consulting with the prison officers about specific rules, we deployed questions on prisoners' age, body weight, and time for sitting and lying indoors (**Table 4**).

### Measurements for Psychological Parameters

The psychological parameters of interest were measured using well-established questionnaires (Table 2). The validated Chinese versions of the questionnaires were employed in the current study (except for the distress tolerance scale, which was translated into Chinese using the back-and-forth translation method).

**Table 2:** Questionnaire description.

**Dimensions**	**Questionnaire**	**Outcomes**	**Literature for validation**
Depression	Patient health questionnaire-9 (PHQ-9)	Higher scores indicate greater depression (range: 0–27 points)	(47)
Anxiety	Generalized anxiety disorder scale (GAD-7)	Higher scores indicate greater anxiety (range: 0–21 points)	(48)
Loneliness	Short-form UCLA loneliness scale (ULS-6)	Higher scores indicate greater loneliness (range: 6–24 points)	(49)
Life satisfaction	Satisfaction with life scale (SWLS)	Higher scores indicate greater life satisfaction (range: 5–35 points)	(50)
Well-being	5-item world health organization well-being index (WHO-5)	Higher scores indicate greater well-being (range: 0–25 points)	(51)
Distress tolerance	Distress tolerance scale (DTS)	Higher scores indicate greater distress tolerance (range: 1–5 points)	(52)

### Data Inclusion

There were 2,572 prisoners in the three studied prisons during the study period, and they were all informed of the investigation. As a result, 326 prisoners voluntarily participated in the survey, but 57 quit the investigation for an infection test, and their questionnaires were left completely blank. Thus, their information was not recorded. Eventually, 269 finished the questionnaire, resulting in a response rate of 10.46% (the minimum response rate defined by the American Association for Public Opinion Research). All of the 269 prisoners who completed the questionnaire were included for analysis.

### Statistical Analysis

Internal reliabilities of the questionnaires were analyzed with Cronbach's alpha. Spearman's correlation analysis was performed to examine the correlations between the measured parameters. To test and quantify the causal pathways in the conceptual framework, we developed a latent variable to represent mental health problems, which was reflected by the observable variables depression and anxiety. Structural equations modeling (SEM) was employed to investigate the causal relationships in the framework. According to Bagozzi and Yi (53), the sample size for the SEM framework should be double the number of variables in the model or more. Therefore, our sample size was appropriate. The goodness of fitting was assessed by the following index: χ^2^-test (*p* > 0.050); χ^2^/df < 2.000; root mean square error of approximation (RMSEA) < 0.080; adjusted goodness of fit index (AGFI) > 0.900; Bentler's comparative fit index (CFI) > 0.900; and Bentler-Bonett normed fit index (NFI) > 0.900. Statistical analysis was performed using SPSS 25.0 and AMOS 21 (SPSS Inc. IL, Chicago, USA). A two-sided *p* < 0.05 was considered statistically significant in the current study.

## Results

### Validation of Questionnaires

As all the questionnaires were applied in a prison population for the first time, the internal consistency was tested. Ideal internal consistency (Cronbach's alphas) was observed for all questionnaires (Table 3), which allowed further analysis.

**Table 3:** Internal consistency of the questionnaires.

			**Questionnaire**			
	**PH-9**	**GAD-7**	**ULS-6**	**DTS**	**SFWL**	**WHO-5**
Dimension	Depression	Anxiety	Loneliness	Distress tolerance	Life satisfaction	Well-being
Cronbach's α	0.896	0.915	0.808	0.847	0.880	0.931

### Characteristics of the Subjects

Only part of the prisoners provided information on age and their time indoors (Table 4). Three percent of the prisoners reported no greenspace viewing through windows, 29.4% reported that a bit greenspace was visible, 48.0% reported that some greenspace was visible, and 19.7% reported that much greenspace was visible.

**Table 4:** Prisoners' characteristics.

**Item**	***N***	**Category**	**Percentage**	**Mean (SD)**
Age (year)	229			34.45 (8.09)
Weight (kg)	229			63.83 (10.86)
Sitting (h/day)	216			6.83 (2.95)
Lying (h/day)	269			9.50 (0)
Visibility	8	Invisible	3.0%	
	79	A bit	29.4%	
	129	Some	48.0%	
	53	Much	19.7%	
Frequency (time/day)	269			3.91 (3.21)
Duration (min/time)	269			5.68 (6.20)
Depression	269			6.04 (5.25)
Anxiety	269			4.13 (4.42)
Loneliness	269			10.50 (4.21)
Distress tolerance	269			2.91 (0.52)
Life satisfaction	269			14.13 (6.46)
Well-being	269			13.15 (6.98)

### Correlation Between Measured Parameters

**Figure 2:**
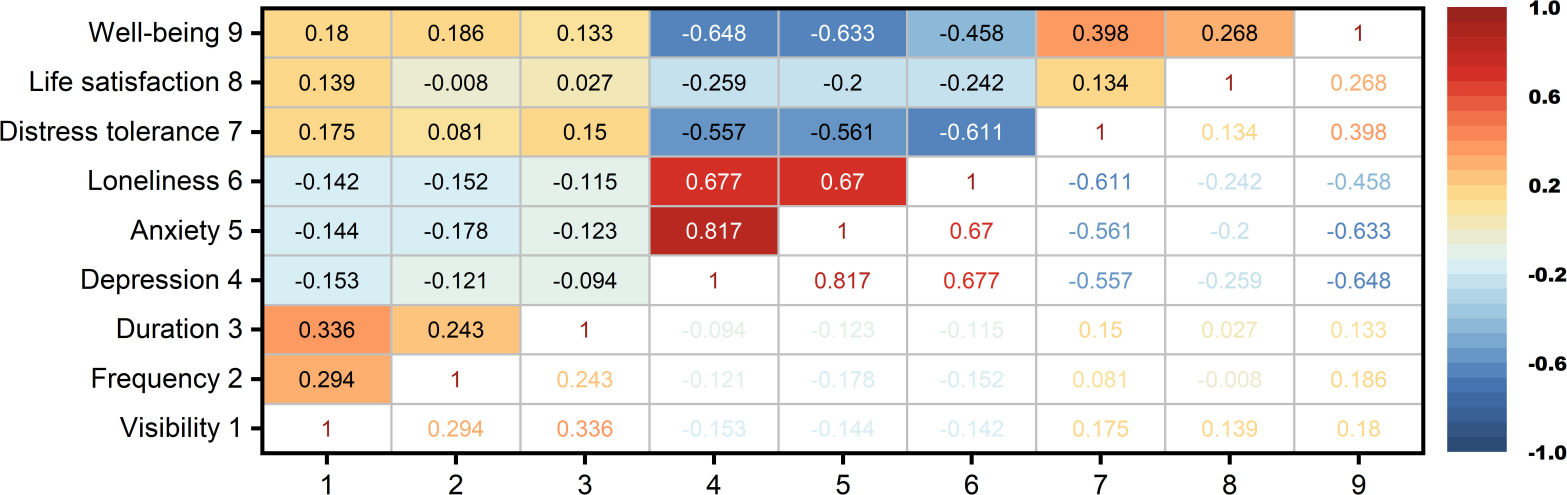
Spearman's correlation analysis for the measured parameters. Numbers in the cells indicate Spearman's ρ.

The visibility of nature statistically significantly correlated with all measured parameters (*p* < 0.05). The frequency statistically significantly correlated with duration (*p* < 0.001), depression (*p* < 0.047), anxiety (*p* = 0.003), loneliness (*p* = 0.013), and well-being (*p* = 0.002). The duration statistically significantly correlated with anxiety (*p* = 0.044), distress tolerance (*p* = 0.014), and well-being (*p* = 0.029; Figure 2).

### The Final SEM Model

**Figure 3:**
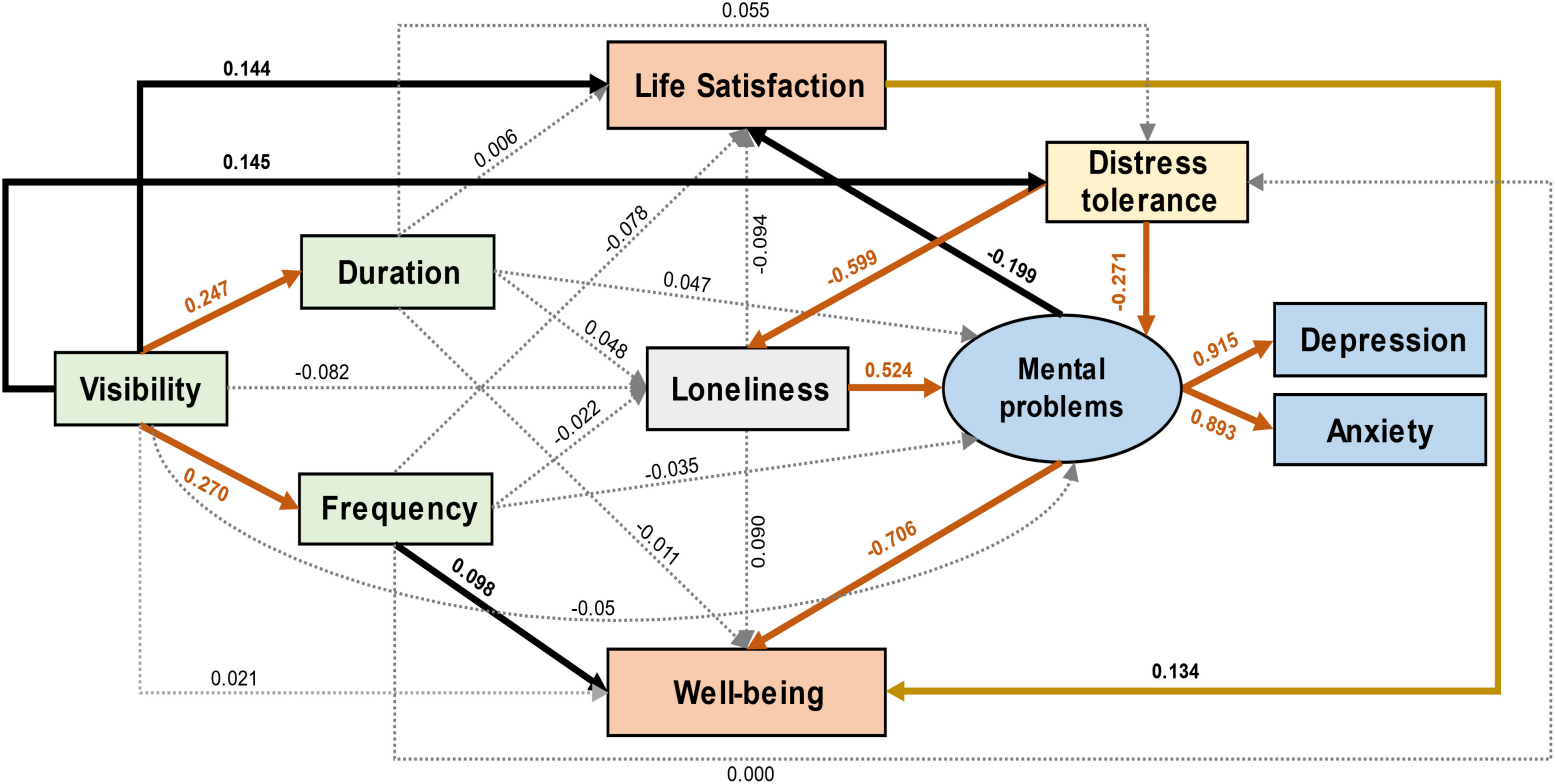
The final SEM model demonstrates standardized effects between variables. The black solid lines indicate *p* < 0.05, the yellow solid line indicates *p* < 0.01, the red solid lines indicate *p* < 0.001, and the dotted lines indicate *p* > 0.05.

**Figure 4:**
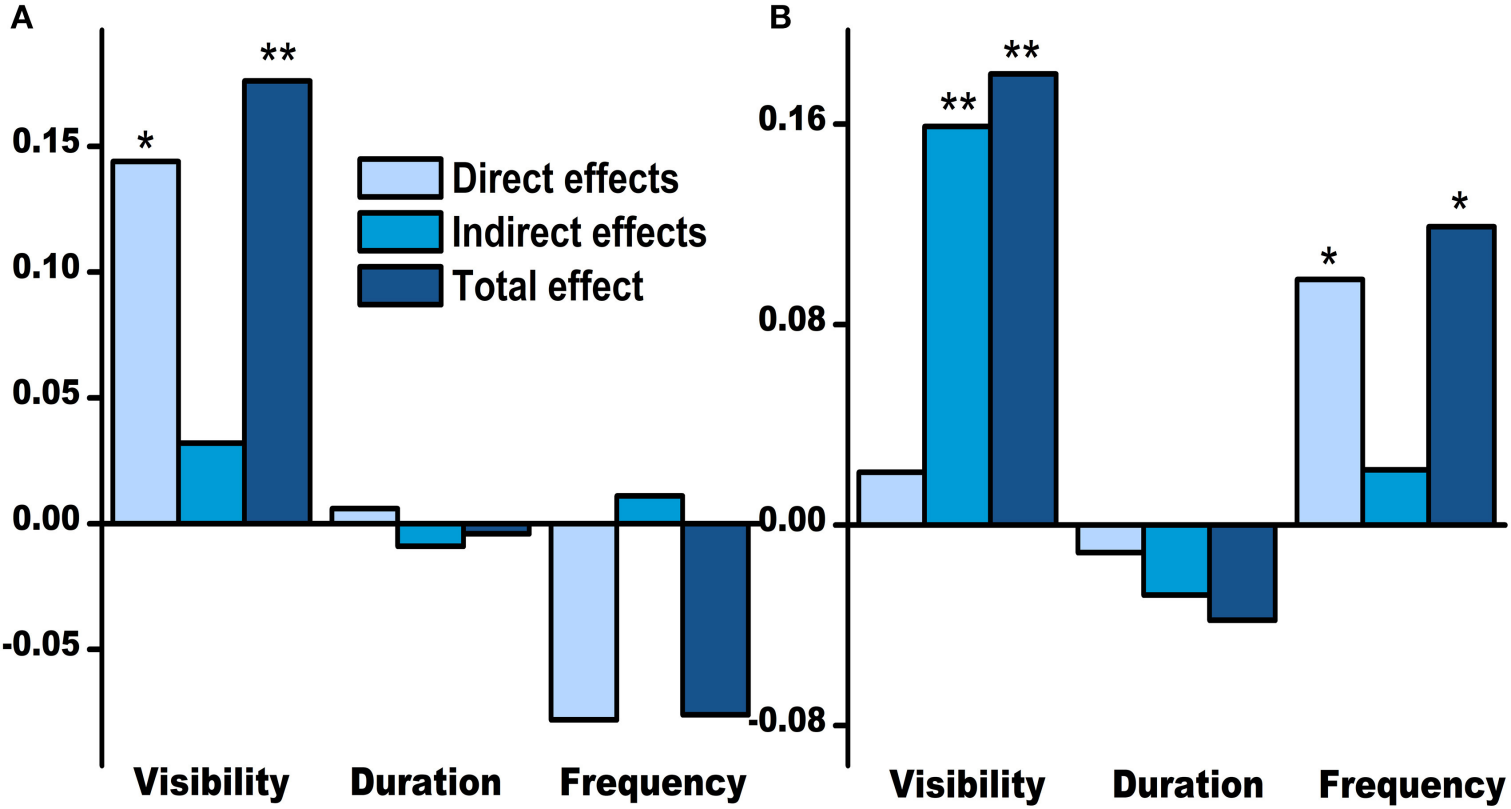
The standardized total, direct, and indirect effects of nature exposure-related variables on prisoners' life satisfaction **(A)** and well-being **(B)**. *p < 0.05; **p < 0.01.

The initial model had an acceptable fit to the data (χ^2^ = 15.676, df = 9, *p* = 0.074, χ^2^/df = 1.742, NFI = 0.982, AGFI = 0.938, CFI = 0.992, and RMSEA = 0.053). Therefore, all pathways in the conceptual framework were tested and quantified (Figure 3). The SEM model indicated that visibility of nature had a statistically significant positive effect on life satisfaction (standardized total effect = 0.176, *p* = 0.005), mainly *via* direct effects (standardized direct effect = 0.144, *p* = 0.029) (Figure 4). On the other hand, visibility of nature also showed a statistically significant positive effect on well-being (standardized total effect = 0.180, *p* = 0.003), but mainly *via* indirect effects (standardized indirect effect = 0.159, *p* = 0.004) (Figure 4). The visibility of nature was observed to significantly promote the duration and frequency of viewing nature through windows (standardized direct effect = 0.247 and 0.270, respectively, *p* < 0.001) (Figure 3). However, only the frequency of visiting nature through windows was observed to have a direct positive effect on well-being (standardized direct effect = 0.098, *p* = 0.038), and the duration of viewing nature through windows did not show a statistically significant effect on any variables (*p* > 0.05; Figure 3).

Although the visibility of nature mainly affected life satisfaction *via* direct effects, there were several preset conceptual indirect pathways with statistical significance. The visibility of nature also exerted a significant positive indirect affect on life satisfaction *via* increasing distress tolerance that directly decreased mental health problems and indirectly decreased them by reducing loneliness (Table 5). Regarding well-being, the visibility of nature increased well-being by increasing the frequency of viewing nature through windows or directly increasing life satisfaction. Meanwhile, the same indirect pathways of visibility—life satisfaction were also found from the visibility to well-being. Two longer routes comprised the above two pathways, but the effects were subtle (Table 5).

**Table 5:** Statistically significant indirect pathways from visibility of nature to prisoners' life satisfaction and well-being.

**Pathways**	**Estimate**	***p***
Visibility → Distress tolerance → Mental problem → Life satisfaction	0.008	0.031
Visibility → Distress tolerance → Loneliness → Mental problem → Life satisfaction	0.009	0.034
Visibility → Life satisfaction → Well-being	0.019	0.029
Visibility → Frequency → Well-being	0.026	0.020
Visibility → Distress tolerance → Mental problem → Well-being	0.028	0.030
Visibility → Distress tolerance → Loneliness → Mental problem → Well-being	0.032	0.043
Visibility → Distress tolerance → Mental problem → Life satisfaction → Well-being	0.001	0.026
Visibility → Distress tolerance → Loneliness → Mental problem → Life satisfaction → Well-being	0.001	0.026

## Discussion

### Visibility of Nature and Human Behaviors

The current study suggests that higher visibility of nature through windows may promote both frequency and duration in viewing nature through windows, underlining the causal relationship between the two dose-response variables and the visibility of nature. This finding implies that the view of nature may be attractive in a prison environment. Such attraction could be related to the dull and lonely prison lives during the pandemic. Although the investigated Chinese prisoners were less isolated than prisoners from other countries, they also spent more than 10 h sitting or lying indoors (Table 4), which implies considerable isolation time. Unlike the quarantined non-imprisoned people who can use smartphones and networks for recreation (54), Chinese prisoners cannot use electronic equipment. Therefore, prisoners may resort to nature viewing for recreation and relaxation. According to recent studies, people might engage in nature for enjoyment and recreation, and viewing nature may help people reduce physiological and psychological stress (55, 56). Therefore, these benefits may promote prisoners' visual contact with nature through windows. Since the nearby greenspaces may offer major natural elements for window views (21), increasing greening around the prisons may enhance the visibility of nature and benefit prisoners.

### Window View of Nature and Mental Health Problems

The current study identified indirect adverse pathways from the visibility of nature through windows to mental health problems represented by depression and anxiety symptoms. The results support a previous study where the views of greenspaces from home were associated with a lower risk of anxiety and depression (22). A later study has also confirmed the same benefits for isolated people during the COVID-19 pandemic (21), which underlines the values of nature exposure for the isolated population. Since prisoners are facing severer isolation during the pandemic, strategies such as contactless social engagement and communication are recommended to alleviate mental health problems (57). However, these strategies require more labor power and material resources. By comparison, nature exposure may be a convenient and low-cost alternative.

In terms of the pathways from the visibility of nature to mental health problems, we found that distress tolerance was a mediator linking the visibility of nature and mental health problems. This result is similar to a previous study, where residential greenspace predicted emotional resilience in children (58). These findings may partially explain the observed benefits of nature exposure for mental health (26, 59, 60). Moreover, our results indicate that loneliness was a mediator of mental health problems, and it is negatively affected by distress tolerance, which may partially explain the previously observed negative association between loneliness and nature views through windows (21). Since loneliness has become a common problem during the pandemic, these results also imply the value of the green window views in improving the mental health of people who are quarantined alone.

### Prisoners' Satisfaction With Life and Well-Being

In the current study, we found that the visibility of nature mainly affected life satisfaction *via* direct effects. The result concerning life satisfaction broadly concurs with those of Chang et al. (38), who found a significant positive correlation between nature views and life satisfaction. However, Chang et al. (38) recognized that they could not eliminate the possibility that wealthier people may have higher life satisfaction and choose to live in neighborhoods with better access to natural spaces. By comparison, the visibility of nature in the current study is only determined by the physical location of prisoners' cells. Therefore, our study may confirm the causal relationship between nature view and life satisfaction. Specifically, the increased life satisfaction was partially influenced by the visibility of nature in the prison environment. In terms of well-being, we found that visibility of nature displayed a significant and positive effect on prisoners' well-being. This finding is similar to those of Gilchrist et al. (39), who found that greenspace views at workplaces were associated with higher levels of employee well-being. These observed benefits about window views are generally in line with previous findings on existing nature-based projects (61), indicating that indirect nature exposure may be considered a potential public intervention for mental health, even in isolated prisoners.

In general, the current study suggests that viewing nature through windows may improve prisoners' lives during the COVID-19 pandemic. Previously, horticultural work has been used as a nature-based intervention for prisoners, and it has been found to improve the mental well-being of prisoners (18, 62). However, such direct physical access to nature is currently not practical due to the epidemic. Therefore, alternative solutions are needed, especially the methods that can be applied in prisons. Based on our findings, increasing greening within and around prisons as well as improving the structure of windows are helpful. Besides, offering prisoners who are quarantined with better greenery views may help reduce mental illness and related consequences (8). On the other hand, though viewing nature was found beneficial, increasing the visibility of nature with extra greening may be costly. In this context, some other methods can be considered. For example, a recent effort uses virtual reality technology to deliver benefits of nature exposure for incarcerated people, which may be an option for mental healthcare in prison systems (56, 63, 64). Before the pandemic, a study indicated that wall-images of natural environments could make some prisoners feel calm and clear their minds (19). This method is more economical and worth trying in prisons during the special period.

### Limitations

Only male prisoners were included in the current study. Thus, our findings may not be extrapolated to female counterparts, and the situations in female prisoners still need investigation. Due to our limited experimental conditions, visibility of nature was measured using self-reports, and objective measures of the visible environments such as distance from greenspaces, point of view, and rate of residential vegetation greenness were not available. In addition, the current study only considered the visibility of nature *via* a window, while the features of visible natural elements such as plant type or greenspace type were not investigated. Previous studies suggest that vegetation and environment types may be related to subjective feelings and psychological outcomes, highlighting the necessity of further studies (39, 65). Finally, only a small number of prisoners participated in our investigation. The low response rate may limit the reliability of our results. For example, we found that only 3.0% of participants reported no nature was in sight, lower than expected. As our study was introduced as a survey on environmental health, those prisoners who benefited from nature views might be more likely to participate in our investigation. This bias may make us overestimate the benefits of viewing nature *via* windows. Accordingly, future research may require better incentives to promote participation.

## Conclusions

The current study examined the pathways from nature exposure through windows to prisoners' life satisfaction and well-being. We found that the visibility of nature promoted the frequency and duration of viewing nature through windows. However, only the frequency directly affected well-being, while the duration showed no distinctive effect on any measured variables. Generally, the visibility of nature through windows may promote distress tolerance, and thus reduce loneliness and mental health problems. As a result, these mental health benefits may eventually benefit prisoners' life satisfaction and well-being. Based on our findings, policymakers may consider nature-based solutions such as visual contact with nature to promote prisoners' lives.

## Data Availability Statement

The raw data supporting the conclusions of this article will be made available by the authors, without undue reservation.

## Ethics Statement

The studies involving human participants were reviewed and approved by the Ethics Review Board of Southwest University. The patients/participants provided their written informed consent to participate in this study.

## Author Contributions

HL designed this study and draft the manuscript. XZ and HL carried out the experiment. GZ supervised the project. HL and YC processed the data. YC, CY, and XC revised the manuscript. GZ and YC made critical revisions to this paper. All authors have read and approved the final manuscript.

## Funding

This research was funded by the Fundamental Research Funds for the Central Universities (SWU1909025).

## Conflict of Interest

The authors declare that the research was conducted in the absence of any commercial or financial relationships that could be construed as a potential conflict of interest.

## Publisher's Note

All claims expressed in this article are solely those of the authors and do not necessarily represent those of their affiliated organizations, or those of the publisher, the editors and the reviewers. Any product that may be evaluated in this article, or claim that may be made by its manufacturer, is not guaranteed or endorsed by the publisher.
